# Biventricular Takotsubo Cardiomyopathy Secondary to COVID-19

**DOI:** 10.7759/cureus.36153

**Published:** 2023-03-14

**Authors:** Bradley Casey, Gregory Chen, Amol Bahekar, Divyang Patel, Raviteja Guddeti

**Affiliations:** 1 Internal Medicine, Cape Fear Valley Medical Center, Fayetteville, USA; 2 Cardiology, Cape Fear Valley Medical Center, Fayetteville, USA; 3 Cardiovascular Medicine, Creighton University School of Medicine, Omaha, USA

**Keywords:** cardiac chest pain, biventricular failure, pea arrest, takotsubo cardioyopathy, covid 19

## Abstract

Takotsubo cardiomyopathy (TCM) is a heart failure syndrome characterized by acute and transient dysfunction of the apical segment of the left ventricle. Since the emergence of coronavirus disease 2019 (COVID-19), caused by the severe acute respiratory syndrome coronavirus 2 (SARS-CoV-2), the diagnosis of TCM has increased in prevalence. Here we present an intriguing case of a patient who initially presented to the hospital with respiratory failure and received a diagnosis of COVID-19. During the patient's hospital course, he was also diagnosed with biventricular TCM and subsequently experienced complete resolution of TCM before discharge. Providers should be cognizant of the potential cardiovascular complications of COVID-19 and consider those heart failure syndromes, including TCM, could be causing some of the respiratory dysfunction in these patients.

## Introduction

First described in 1990 and 1991 by Sato and his colleagues, takotsubo cardiomyopathy (TCM) is a heart failure syndrome characterized by its acute and typically reversible nature [1,2]. The label "takotsubo" refers to the similarity in appearance of the left ventricle at end-diastole to the pots used by Japanese fishermen in the fish markets of Hiroshima to capture octopus [2]. Frequently preceded by some major physical or emotional stressor, TCM mimics acute coronary syndrome and is characterized by transient systolic and diastolic dysfunction of the left ventricle, wall motion abnormalities, and elevated cardiac biomarkers [3]. While the precise manner of causation of Takotsubo syndrome is unknown, the sudden release of stress hormones, including dopamine, epinephrine, and norepinephrine, is thought to be the reason for cardiac stunning [4]. The clinical range of Coronavirus disease 2019 (COVID-19) includes an asymptomatic infection or mild upper respiratory infection up to acute respiratory failure, shock, and even death [5]. Infection can also lead to cardiovascular sequelae, with the involvement of heart failure a signifier of worse clinical outcomes [5]. In patients who develop COVID-19, it is conceivable that the profound hypoxia associated with acute respiratory distress syndrome may trigger a surge of catecholamines, leading to myocardial stunning and, ultimately, TCM [5]. When both ventricles are involved, patients will typically have a more severe clinical course, with the prevalence of this complication estimated to be less than 0.5% of all anatomical variants of TCM [1]. Here we present a rare case of a patient found to have biventricular (BV) TCM secondary to COVID-19.

## Case presentation

This is a 52-year-old Caucasian male with a past medical history of tobacco abuse who presented to the emergency department with a chief complaint of shortness of breath and generalized weakness. The patient arrived via ambulance with an oxygen saturation of 88% on a 3L nasal cannula. The patient described shortness of breath as progressive over the prior seven days, a sensation of the inability to take a full, deep breath, an inability to ambulate 25 feet, an inability to sleep lying flat, and coughing up green sputum. The patient also endorsed lightheadedness and generalized weakness for the prior three days. Initial vitals included temperature and blood pressure within normal limits, a respiratory rate ranging between 30 and 37, and a sinus tachycardia heart rhythm/rate ranging between 110 and 122 beats per minute. Initial lab work can be seen in Table 1.

**Table 1:** Patient's initial blood work upon arrival to the emergency department

Laboratory Tests	Reference Range	Patient's Admission Lab Values
Lactic Acid	0.5 to 2.0 mmol/L	2.1 mmol/L
C-Reactive Protein	Less than 5 mg/L	167 mg/L
Sodium	136 to 145 mmol/L	128 mmol/L
White Blood Cell Count	4.5-12.5 x 10*3/uL	24.2 x 10*3/uL
Platelets	150-450 x10*3/uL	78 x 10*3/uL
High Sensitivities Troponin	2 to 20 pg/ml	31 pg/ml
Repeat 3-hour High Sensitivity Troponin	2 to 20 pg/ml	34 pg/ml
COVID-19 SARS-CoV-2 PCR	Negative	Positive

**Figure 1:**
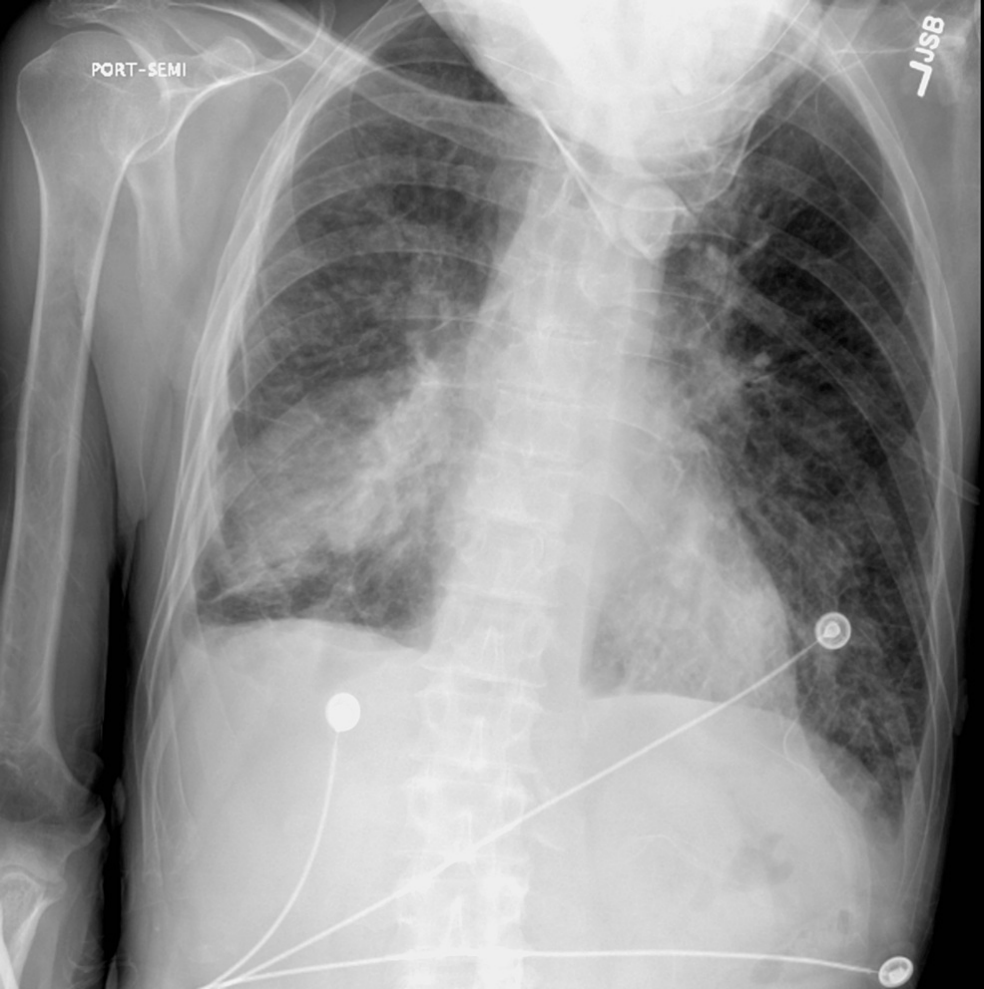
Chest X-ray showing right lower lobe airspace consolidation with interval development of findings suggestive of vascular congestion with congestive heart failure.

**Figure 2:**
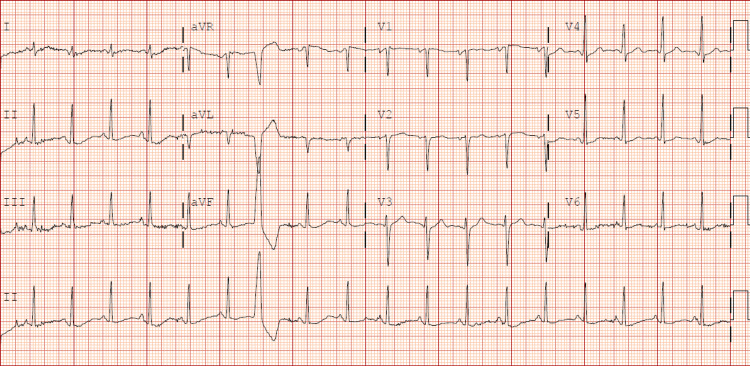
Sinus tachycardia with a rate of 112, premature ventricular complex

Chest X-ray demonstrated right lower lobe airspace consolidation with interval development of findings suggestive of vascular congestion with congestive heart failure versus pneumonia (Figure 1). A CT scan of the chest was completed and showed no evidence of pulmonary embolism, bilateral nodular infiltrates, and areas of consolidative density in the right middle lobe and right lower lobe. Due to the patient's chest X-ray and worsening respiratory status, a transthoracic echo was obtained, which showed normal LV size and wall thickness with severe global apical hypokinesis, a left ventricular ejection fraction of approximately 25% to 30%, trace tricuspid regurgitation, and mild to moderate RV enlargement (Video 1). Initial EKG (Figure 2) showed sinus tachycardia with a rate of 112 and ventricular premature complex. Upon comparison to an EKG completed four months prior, no significant changes were found. Due to increasing respiratory distress, the patient was intubated and transferred to the intensive care unit (ICU). 

**Video 1:** This is the initial transthoracic echocardiogram performed upon the patient's arrival to the emergency department, which showed normal left ventricular size and wall thickness with severe global apical hypokinesis, a left ventricular ejection fraction of approximately 25% to 30%, trace tricuspid regurgitation, and mild to moderate right ventricular enlargement.

**Figure 3:**
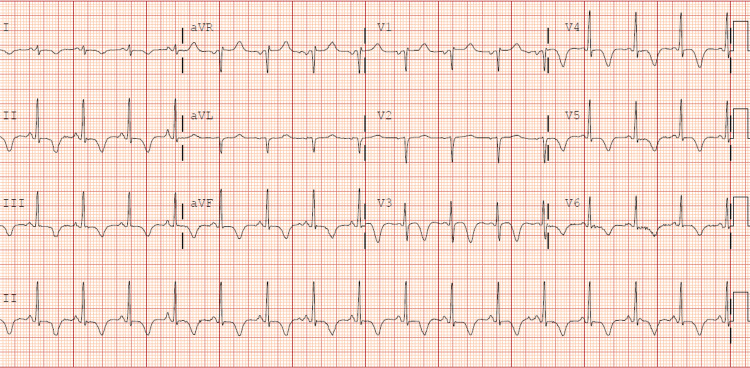
Second EKG during a chest pain episode. Sinus rhythm with a rate of 95, QTc 499, and T-wave inversion seen in leads II and III, aVF, V3, V4, V5, and V6

Overnight in the ICU, the patient self-extubated, resulting in a pulseless electrical activity (PEA) arrest. The patient only required one round of advanced cardiac life support (ACLS) before the return of spontaneous circulation (ROSC) was achieved, and he was reintubated. Over the next four days, the patient's respiratory status improved, and he was extubated. He was eventually transferred to the medicine-telemetry floor. At the time of his transfer out of the ICU on day nine, the patient was complaining of midsternal, sharp, non-radiating chest pain that scored an eight out of 10 on the pain scale. Nothing made it better, and he could not describe anything that made it worse. He reported that this pain was different from the pain he was experiencing from the chest compressions. At this time, the patient had been transitioning off of bilevel-positive airway pressure for the past 24 hours, and he was becoming more anxious about having to put the mask back on. One of the initial differentials for his chest pain included the patient being overstimulated and having a panic attack due to his increased respiratory rate. The repeat high-sensitivity troponin at that time was 28 pg/ml (normal: 2-20 pg/ml). A repeat EKG (Figure 3) showed sinus rhythm with a rate of 95, QTc of 499, and T-wave inversion seen in leads II and III, aVF, V3, V4, V5, and V6 that were not seen on the initial EKG (Figure 2). The patient also had a repeat CT scan with contrast that was negative for pulmonary embolism. At this time, cardiology was consulted, and a left heart catheterization was performed (Videos 2, 3). Cardiac catheterization showed patent coronary arteries with calcified nonobstructive coronary artery disease, 30% to 40% mid-left circumflex stenosis, 40% to 50% proximal mid-RCA stenosis, and right dominant circulation. Repeat TTE was performed the day after a cardiac catheterization, revealing normal LV systolic function, an ejection fraction greater than 55%, an impaired relaxation pattern of LV diastolic filling, and mild concentric left ventricular hypertrophy (Videos 4, 5). A total of 12 days passed between the initial TTE and the subsequent TTE, which showed a resolution of biventricular dysfunction. The patient had a prolonged hospital stay due to his consistent requirement for bilevel-positive airway pressure, but eventually, he was comfortable on room air. After cardiac catheterization and a repeat TTE, the diagnosis of biventricular takotsubo secondary to COVID-19 was made by cardiology. A repeat EKG before hospital discharge (Figure 3) showed a sinus rhythm of 80 bpm with a resolution of the inverted T-waves. The patient's oxygen saturation eventually improved to 97% on room air, and he was discharged home.

**Video 2:** Left heart catheterization. Left anterior descending artery showing minor luminal irregularities, Diagonal 1 and Diagonal 2 are widely patent, and the circumflex is mildly calcified with 30%-40% midsegment stenosis.

**Video 3:** Right coronary artery catheterization showed calcification up to 40%-50%, mostly in the proximal and mid segments.

**Video 4:** Cardiac echocardiogram that was performed prior to discharge. This was taken 12 days after the original. Left ventricular systolic function is normal with ejection fraction greater than 55 percent.

**Video 5:** This is the parasternal long axis view, which shows a left ventricular ejection fraction greater than 55 percent and systolic function back to normal.

## Discussion

While isolated LV involvement in TCM is the most common variant, a few other variants do occur [6]. Right ventricular (RV) involvement is one such variant garnering recognition [6]. Joseph Doako et al. determined from a literature review that biventricular involvement substantially impacts patient prognosis, morbidity, and outcomes related to its impact on hospitalization length and hemodynamic instability [6]. In the United States, the estimated incidence of TCM is 0.02% of all hospitalizations and 2% of all acute coronary syndrome presentations [3]. Computer-assisted SCOPUS or MEDLINE searches of the literature for the terms apical ballooning, ampulla cardiomyopathy, tako-tsubo cardiomyopathy, and takotsubo cardiomyopathy between 1989 and December 2007 indicated an exponentially increasing frequency of publications [7]. The prognosis of TCM is usually favorable; however, detrimental complications such as free wall rupture can occur [6]. Kazufumi Tsuchihashi et al. studied 88 patients who met the criteria of transient LV apical ballooning, no significant angiographic stenosis, and no known cardiomyopathies. Of those 88 patients, chest pain occurred in 59, dyspnea in six, atrial fibrillation in six, pulmonary edema in 19, and cardiogenic shock in 13 patients [8].

To diagnose TCM, we use the modified Mayo Clinic criteria: 1) absence of obstructive coronary artery disease on angiography; 2) transient dyskinesis, hypokinesis, and akinesis of the left ventricle mid-segments with or without apical involvement; 3) ECG evidence of ST-segment elevation and/or T-wave inversion; 4) modest elevation of troponin levels; 5) absence of myocarditis or pheochromocytoma [4]. Our patient met these requirements: non-obstructive CAD did not explain the degree of his wall motion abnormalities; he had a modest elevation in troponin; a T-wave inversion on the EKG; and wall motion abnormalities on the TTE. And finally, this patient also experienced resolution of the wall motion abnormality by the time of discharge, as demonstrated in Videos 1, 2.

The exact underlying etiology of TCM is not well understood. As discussed above, the most plausible cause is the sudden release of stress hormones [4]. Several reports in the literature describe the numerous cardiovascular complications of COVID-19 infection, including acute myocardial infarction, myocarditis, cardiomyopathy, arrhythmias, and venous thromboembolism [5]. It is believed that acute illness with COVID-19 may trigger a surge of catecholamines, leading to TCM [5]. The SARS-CoV-2 virus receptor, angiotensin-converting enzyme 2 (ACE2), is expressed in myocytes and vascular endothelium and could be an underlying mechanism for direct myocardial injury in COVID-19 disease [5].

Management of TCM is currently empirical and supportive, although it has been emphasized that such non-specific therapies are consequent to its still-elusive pathophysiology [9]. There are no randomized trials or even expert consensus to guide medical therapy, and management recommendations are based on a current understanding of the pathophysiology and natural history of the disease [10]. Treatment includes supportive care, targeting of the precipitating trigger (when known), beta-blockade, inhibitors of the renin-angiotensin system, and consideration of systemic anticoagulation in all patients [10]. Left ventricular function is expected to return to normal regardless of the initiation of early therapy [10]. Our patient experienced complete resolution of his biventricular cardiomyopathy before discharge, hypothesized to be a result of the resolution of the COVID-19 catecholamine surge.

## Conclusions

Reversible TCM may occur in the setting of a COVID-19 infection with elevated cardiac biomarkers and an abnormal ECG. Here we present an interesting case of COVID-19 resulting in BV TCM. COVID-19 can predispose patients to develop TCM, as the virus plays numerous roles in physical stress during the acute phase of the infection. Due to the common respiratory symptoms that COVID-19 causes, TCM could easily be missed in the initial presentation of COVID-19. In patients presenting with COVID-19 and any symptoms consistent with heart failure, providers need to have a low threshold for obtaining an echocardiogram.
